# Supplementary material for: Overexpression of MdARD4 Accelerates Fruit Ripening and Increases Cold Hardiness in Tomato
Source: Int J Mol Sci. 2020 Aug 27;21(17):6182. doi: 10.3390/ijms21176182 (PMC7503420; doi:10.3390/ijms21176182)
Supplement: Supplementary file 1 [file ijms-21-06182-s001.pdf]

Table S1. Primers used in our study.

Gene	Primer	Sequence (5′–3′)
**Cloning and vector construction**		
MdARD1	*MdARD1*-S	ATGGTTGCCACCCAAAAGGATC
	MdARD1-A	TTATGCCGCAGCATTAACTGCAT
MdARD2	*MdARD2*-S	ATGGCTGCCTCCCAAAGGGATC
	MdARD2-A	CTATGCCGCAGCGTTAACTGCAT
MdARD4	*MdARD4*-S	ATGGCGATGGAGGCATGGTT
	MdARD4-A	TTAATGAGCTTTTAATGCCACTCC
MdARD4	pCambia2300-*MdARD4*-S	TCCCCCGGGATGGCGATGGAGGCATGGTT
	pCambia2300-*MdARD4*-A	CGAGCTCTTAATGAGCTTTTAATGCCACTCC
MdARD1	GFP-*MdARD1*-S	GGGGACAAGTTTGTACAAAAAAGCAGGCTTCATGGTTGCCACCCAAAAGGATC
	GFP-*MdARD1*-A	GGGGACCACTTTGTACAAGAAAGCTGGGTTTGCCGCAGCATTAACTGCAT
MdARD2	GFP-*MdARD2*-S	GGGGACAAGTTTGTACAAAAAAGCAGGCTTCATGGCTGCCTCCCAAAGGGATC
	GFP-*MdARD2*-A	GGGGACCACTTTGTACAAGAAAGCTGGGTTTGCCGCAGCGTTAACTGCAT
MdARD4	GFP-*MdARD4*-S	GGGGACAAGTTTGTACAAAAAAGCAGGCTTCATGGCGATGGAGGCATGGTT
	GFP-*MdARD4*-A	GGGGACCACTTTGTACAAGAAAGCTGGGTTATGAGCTTTTAATGCCACTCC
**Quantitative real-time PCR**		
MdARD1	RT-*MdARD1*-S	ATGGTTGCCACCCAAAAGGATC
	RT-*MdARD1*-S	ATAGTTATCAGCATCTAGACGCCAA
MdARD2	RT-*MdARD2*-S	ATGGCTGCCTCCCAAAGGG
	RT-*MdARD2*-A	GTAGTTATCAGCATCTAGATGCCAG
MdARD4	RT-*MdARD4*-S	ATGGATTTGCTTGACATATGCC
	RT-*MdARD4*-A	GATCACCGGCCTTAATCCAAAT
MdEF-1α	RT-*MdEF-1α*-S	ATTCAAGTATGCCTGGGTGC
	RT-*MdEF-1α*-A	CAGTCAGCCTGTGATGTTCC
MdActin	RT-*MdActin*-S	TGACCGAATGAGCAAGGAAATTACT
	RT-*MdActin*-A	TACTCAGCTTTGGCAATCCACATC
SlPSY1	RT-*PSY1*-S	AGAGGTGGTGGAAAGCAA
	RT-*PSY1*-A	TCTCGGGAGTCATTAGCAT
SlPDS	RT-*PDS*-S	GCTTTACCCGCTCCTTTA
	RT-*PDS*-A	ACCTTGCTTTCTCATCCA

SlZDS	RT-*ZDS*-S	GGTGGGTGCTGAAAAAAAT
	RT-*ZDS*-A	GGAAAGCGGAAATCAAGTT
SlACS2	RT-*ACS2*-S	GAAAGAGTTGTTATGGCTGGTG
	RT-*ACS2*-A	GCTGGGTAGTATGGTGAAGGT
SlACO3	RT-*ACO3*-S	CAAGCAAGTTTATCCGAAAT
	RT-*ACO3*-A	CATTAGCTTCCATAGCCTTC
SlACO1	RT-*ACO1*-S	ACAAACAGACGGGACACGAA
	RT-*ACO1*-A	CTCTTTGGCTTGAAACTTGA
SlRIN	RT-*RIN*-S	GGAACCCAAACTTCATCAGA
	RT-*RIN*-A	TTGTCCCAAATCCTCACCTA
SlE4	RT-*E4*-S	AGGGTAACAACAGCAGTAGCA
	RT-*E4*-A	CCCAACCTCCGTCTTCAC
SlE8	RT-*E8*-S	GGCACCATTCAACATACCG
	RT-*E8*-A	CTTTCACCGAAGAAGCACG
SlERF1	RT-*ERF1*-S	TTTTAGTATCGGATGGACG
	RT-*ERF1*-A	GGCGGAGAAACAGAAGTA
SlCAC	RT-*CAC*-S	CCTCCGTTGTGATGTAACTGG
	RT-*CAC*-A	ATTGGTGGAAAGTAACATCATCG
SlActin	RT-*Actin*-S	CAGCAGATGTGGATCTCAAA
	RT-*Actin*-A	CTGTGGACAATGGAAGGAC
